# Tongue Malacoplakia: a case report

**DOI:** 10.1016/S1808-8694(15)30560-7

**Published:** 2015-10-19

**Authors:** Sérgio Almeida Pinheiro Chagas, Maurício Buzelin Nunes, Rodrigo Tadeu de Puy e Souza, Wanderson Marques da Silva

**Affiliations:** 1 Pathologist, pathologist of the Anatomic Pathology Unit of the Santa Casa de Belo Horizonte/MG; 2 Specialist in pathology, pathologist, assistant at the Anatomic Pathology Unit of the Santa Casa de Belo Horizonte/MG; 3 Medical course at UFMG, specializing in pathology - graduate program of the Faculdade de Ciências Médicas da Santa Casa de Belo Horizonte/MG; 4 Undergraduate of the biomedicine course, UNIFENAS-BH. Intern at the Anatomic Pathology Unit of the Santa Casa de Belo Horizonte/MG. Laboratório de Anatomia Patológica da Santa Casa de Misericórdia de Belo Horizonte/MG

**Keywords:** tongue, malacoplakia

## INTRODUCTION

Malakoplakia is a rare inflammatory disease of indeterminate etiology, which may involve many organs, and that has no specific symptoms.^1^ The disease was first described in 1902 by Michaelis and Gutmann;^2^, its name come from the Greek malakos = soft and plakos = plaque.^3^ The incidence is higher in female patients and between the fifth and seventh decades of life. This disease may, at times, be associated with immune deficiency, malignancies, or immunosuppressive therapies.^2^

Malakoplakia generally occurs in the genitourinary tract; the bladder is the most commonly involved site.^4^ Five cases of malakoplakia on the tongue have been described in the literature.

## CASE REPORT

H.L.R, aged 60 years, male, sought the Otorhinolaryngology unit complaining of swallowing difficulties because of a lesion in the mouth for the past two years. The physical examination showed that the patient was of normal complexion, hydrated, acyanotic and anicteric. The patient was not using immunosuppressant medication. Videolaryngoscopy revealed a soft brown-yellowish lesion to the right of the base of tongue; it measured about 4 cm. Direct laryngoscopy under general anesthesia was done to remove the lesion for pathology.

**Figure 1:**
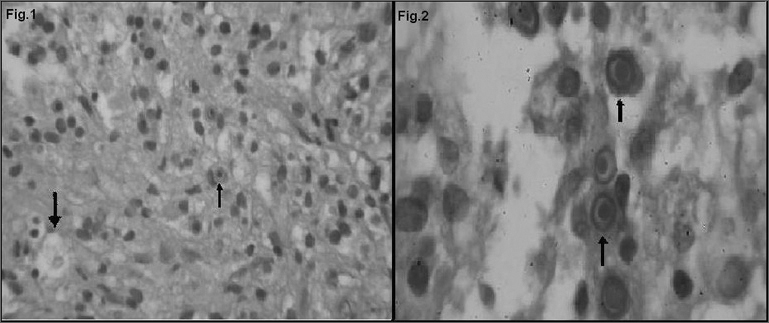
H.E / Figure 2 PAS - Fig. 1: H.E: 40x showing an inflammatory infiltrate, histiocytes with cytoplasmic vacuoles (Von Hansemann cells) and cytoplasmic inclusions. Fig. 2: PAS: Immersion showing cells with cytoplasmic inclusions, the Michaelis-Guttman corpuscles.

Pathology revealed chronic inflammation with frequent histiocytes containing cytoplasmic vacuoles and concentric structures suggesting malakoplakia (Fig. 1). Special histochemical and immunohistochemical staining was done for greater diagnostic precision.

The periodic acid Schiff (PAS) stain showed round basophilic intra-cytoplasmic structures (Michaelis-Gutmann corpuscles) in some histiocytes (Fig. 2). Histiocytes were CD68 positive according to the avidinbiotin peroxidase (ABC) immunohistochemical technique.

These findings suggested the diagnosis of malakoplakia; there was no evidence of malignancy in the material.

Following surgery, the patient progressed favorably, with no intercurrences. Sulfametoxazol-trimetoprim was prescribed as treatment for the lesion. Follow-up in the outpatient unit showed that the patient was well, with no signs of recurrence.

## DISCUSSION

Malakoplakia is a rare inflammatory reaction.^1^ The most common causative agent is Escherichia coli; Proteus vulgaris, Aerobacter aerogenes and Klebsiela pneumoniae, however, have been detected in some cases.^2^ Clinically, patients present a soft brown-yellowish plaque of variable size with a central spot or ulcer and intense peripheral hyperemia.^3^

The diagnosis is made in histopathological exams, which show chronic inflammation consisting of large histiocytes containing positive periodic acid Schiff granules (PAS) also known as Von Hansemann cells, and rounded concentric structures named Michaelis-Gutmann corpuscles.^2^ Immunohistochemistry tests may also be applied; in this case, histiocytes may be CD68 positive.^3^

The pathogenesis is poorly defined. It is possible that intracellular changes in cyclic guanosine monophosphate (cGMP) and cyclic adenosine monophosphate (cAMP) may harm macrophage lysosomes, hindering bacterial degradation and thus making it possible for substances to accumulate in phagolysosomes. The result is increased macrophage cytoplasmic volume with Michaelis-Gutmann corpuscles.^2^, ^4^

Because there are no specific symptoms, malakoplakia should be differentiated from: granular cell tumors, xantogranulomatous inflammation, histiocytosis with massive lymphadenopathy, Langherans cell histiocytosis, undifferentiated carcinoma, atypical bacterial infection, and malignant lymphoma.^5^

Recommended medication for treating malakoplakia include sulfametoxazoltrimetoprim, rifamycin, and the quinolones, because these drugs are able to enter phagocytes and eliminate intracellular bacteria.^2^ Vitamin C and cholinergic drugs, such as betanecol, may also be effective; cholinergic agents increase cGMP and vitamin C decreases cAMP, which reestablishes balance and prevents lysosome injury. These associated medications may be beneficial in the treatment of malacoplakia.^4^

## FINAL COMMENTS

Malakoplakia is a rare inflammatory disease with no specific symptoms; thus, the differential diagnosis is varied. This condition presented in an unusual site in this case (base of tongue); the diagnosis was made using histopathology and confirmed by histochemical and immunohistochemical methods.
